# Feasibility and Safety of Video Endoscopic Inguinal Lymphadenectomy in Vulvar Cancer: A Systematic Review

**DOI:** 10.1371/journal.pone.0140873

**Published:** 2015-10-23

**Authors:** Chai-e Liu, Yan Lu, De-Sheng Yao

**Affiliations:** Department of Gynecologic Oncology, Affiliated Tumor Hospital of Guang Xi Medical University, Nanning, People’s Republic of China; H. Lee Moffitt Cancer Center & Research Institute, UNITED STATES

## Abstract

**Objective:**

To systematically review previous studies and to evaluate the feasibility and safety of video endoscopic inguinal lymphadenectomy (VEIL) in vulvar cancer.

**Methods:**

We conducted a comprehensive review of studies published through September 2014 to retrieve all relevant articles. The PubMed, EMBASE, Web of Science, Cochrane Library, Wan Fang Data and Chinese National Knowledge Infrastructure databases were systematically searched for all relevant studies published in English or Chinese through September 2014. Data were abstracted independently by two reviewers, and any differences were resolved by consensus.

**Results:**

A total of 9 studies containing 249 VEIL procedures involving 138 patients were reviewed. Of the 249 VEIL procedures, only 1 (0.4%) was converted to an open procedure for suturing because of injury to the femoral vein. The range of operative time was 62 to 110 minutes, and the range of estimated blood loss was 5.5 to 22 ml. The range of the number of harvested lymph nodes was 7.3 to 16. The length of hospital stay varied from 7 to 13.6 days across reports. The incidence of lymph node metastasis was 19.7% (27/138), and the recurrence rate was 4.3% (3/70) within 3 to 41 months of follow-up. One or more short-term complications were documented in 18 of 138 (13.0%) patients. Complications after VEIL were observed in 14 (10.13%) patients and in 15 (6.0%) of the VEIL cases, including major lymphocyst formation in 9 (3.6%), lymphorrhea in 2 (0.8%), inguinal wound infection without wound breakdown in 3 (1.2%) and lymphedema in 1 (0.4%).

**Conclusions:**

VEIL appears to be a feasible procedure in the management of vulvar cancer. There may be potential benefits that result in lower morbidity compared to traditional methods, but this has yet to be objectively proven.

## Introduction

Vulvar cancer is a relatively rare gynecologic malignancy with an estimated 4,850 new cases and 1,030 deaths in the US in 2014 according to cancer statistics [1]. Surgery is the cornerstone of treatment for vulvar cancer. Inguinal lymphadenectomy plays an important role in vulvar cancer surgery because the presence of lymph node metastasis is the most important prognostic factor for patients [2–5]. Although this surgery has demonstrated good oncological efficacy, it is plagued with high morbidity such as groin breakdown, infection, lymphocyst formation and lymphedema [6,7]. From the earliest en block dissection to the triple incision technique and preservation of the saphenous vein, modifications have been made to reduce the postoperative complications and decrease the morbidity to some extent without compromising the treatment outcomes [8,9]. However, the benefits have not been as dramatic as expected, and studies have reported relatively high rates of local complications despite these modifications [7,10,11].

Sentinel lymph node mapping is currently a relatively popular approach for decreasing surgical trauma. Its high detection rate and sensitivity has been demonstrated in multiple studies; in addition, this approach is associated with a low inguinal recurrence rate [12–14]. However, sentinel node mapping is primarily applicable to early stage and laterally located disease. In addition, the false negative rate, which has a substantial connection with recurrence, is related to the mapping method, tumor location, and the presence of palpable inguinal nodes [5]. Moreover, an experienced team should perform the sentinel node procedure using combined techniques [15,16]. The laparoscopic method is currently routinely applied in the surgical treatment of a wide range of gynecological diseases, including malignant tumors such as cervical cancer and endometrial cancer [17,18]. This method is associated with a significant reduction in intraoperative blood loss, postoperative morbidity, analgesic requirement, and the length of the hospital stay and recovery period [19–21]. Gynecologic oncologists have also introduced video endoscopic inguinal lymphadenectomy (VEIL) for the management of vulvar cancer, and some preliminary studies have shown that this technique may be feasible and safe. The purpose of this review is to study the feasibility and safety of VEIL for vulvar cancer.

## Materials and Methods

### Literature search strategies

The PubMed, EMBASE, Web of Science, Cochrane Library, Wan Fang Data and Chinese National Knowledge Infrastructure databases were systematically searched for all relevant studies published in English or Chinese through September 2014. The following search terms were used: inguinal lymph node, lymph node excision, inguinal lymphadenectomy, endoscopy, laparoscopy, video-assisted surgery, and video endoscopic inguinal lymphadenectomy; all these terms were also combined with vulvar cancer or vulvar carcinoma. In addition, bibliographies of the included studies were checked manually to determine whether there were additional eligible studies.

### Inclusion and exclusion criteria

Studies were included in the analysis for the following reasons: (1) if the patients had a clinical or pathological diagnosis of vulvar cancer, regardless of age, ethnicity or location; (2) if the patients underwent inguinal lymphadenectomy along with VEIL; and (3) if data on intraoperative blood loss, postoperative morbidity, length of hospital stay, and recurrence rate were assessed as outcomes for measuring the effect of treatment. In the case of duplicate publications, the study by the same author with the most recent results was included. Studies were excluded if patients did not undergo inguinal lymphadenectomy, if included patients were only treated with sentinel lymph node mapping for inguinal lymph nodes, or if the data from VEIL patients could not be obtained.

### Data extraction

A data extraction form was prepared, and the following data were retrieved: first author, year of publication, sample size, International Federation of Gynecology and Obstetrics (FIGO) staging, pathological pattern, operative time and blood loss, number of lymph nodes harvested, length of hospital stay, postoperative complications, duration of follow-up, and recurrence rate. The selection of articles for this review, the decisions regarding inclusion/exclusion and the extraction of data were independently performed by two reviewers, and differences were resolved by consensus.

## Results

### Literature search and study characteristics

**Fig 1:**
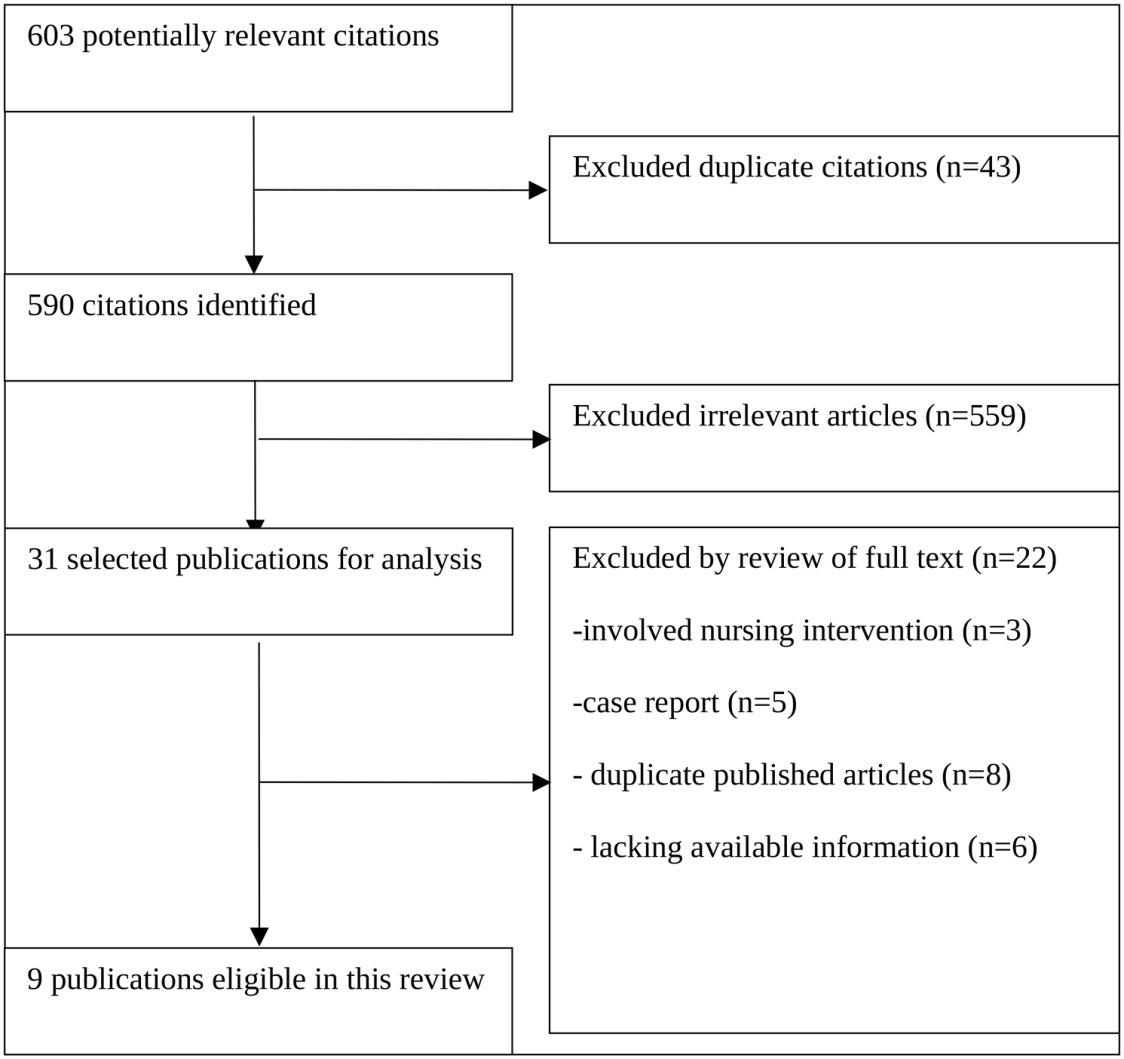
Flowchart of the Selection Process for Studies Included in the Systematic Review.

Using the search strategy described above, a total of 603 potentially relevant studies were initially identified. After careful selection, 31 publications were selected for the analysis. Of these, 3 were excluded because they investigated nursing interventions, 5 were excluded because they were case reports [22–26], 9 were excluded because they lacked available information regarding VEIL in vulvar cancer patients, and 6 were excluded because they were duplicated published articles. A total of 9 eligible studies [27–35] with 249 VEIL procedures in 138 patients were included in the final review. The flow diagram of the study selection is shown in Fig 1, and Table 1 summarizes the key characteristics of the included studies. There was one retrospective case-control study, and the others were retrospective non-control studies. These included 3 articles [27,28,30] that were published in English and 6 [29,31–35] that were published in Chinese. We used the Oxford Centre for Evidence-Based Medicine 2011 Levels of Evidence to assess the quality of the evidence [36]. There were no randomized controlled trials (RCTs) or meta-analyses included in this review, and we used primary statistical methods handling data and combining results of studies.

**Table 1:** Characteristics of the Included Studies.

Author	Publication year	No. pts/ VEIL	Evidence level	Mean age (years)	FIGO stage I/ II/ III	Pathology (SC/AD/other)	Underlying disease (DM/HP)
Wu et al. [27]	2013	10/11	4	47.2(30–68)	6/0/3	7/1/2	-
Mathevet et al. [28]	2002	28/41	4	-	12/12/4	-	-
Cui et al. [29]	2013	15/30	4	51.9(28–66)	6/7/2	15/0/0	1/2
Xu et al. [30]	2011	17/34	4	52.6(33–69)*	8/4/5	15/1/1	1/1
Lu et al. [31]	2012	10/20	4	50.7(31–73)	5/3/2	8/0/2	1/0
Liu et al. [32]	2013	8/13	4	50.2(39–63)	5/1/2	7/0/1	2/2
Xia et al. [33]	2014	13/26	4	48.6(30–71)	6/4/3	10/0/3	-
Li et al. [34]	2014	29/58	4	54.2(37–67)	14/10/5	22/3/4	-
Tang et al. [35]	2012	8/16	4	58.0(48–65)	7/1	8/0/0	-

pts, patients; SC, squamous carcinoma; AD, adenocarcinoma; DM, diabetes mellitus; HP, hypertension;

-, data not available;

*median value.

### Indications for VEIL

All studies showed that VEIL was performed not only for early stage vulvar cancer patients but also for patients with clinical lymph node metastases. In the final surgical pathologic staging, there were 27 patients with stage III disease. In addition, one patient was diagnosed with local recurrence and underwent VEIL with radical vulvectomy; this patient had previously undergone vulvar tumor excision with FIGO-stage IA. A total of 6 studies [29–31,33–35] included bilateral VEIL only, whereas the others [27,28,32] included ipsilateral or bilateral VEIL.

### Operative VEIL parameters (operative time, blood loss, conversion, etc.)

Table 2 shows the various operative parameters. The great saphenous vein with its tributary veins was preserved in all studies. The range of operative time was 62 to 110 minutes (n = 8 studies). The range of estimated blood loss was 5.5 to 22 ml (n = 7 studies). Of the 249 VEIL cases included in the current review, only 1 case (0.4%) was converted to an open procedure for suturing because of an injury to the femoral vein. The other VEIL procedures were performed smoothly without intraoperative complications. The range of the average/median number of harvested lymph nodes was 7.3 to 16 (n = 9 studies). Wu et al. [27] reported that in their later cases, the number of lymph nodes (12–18) excised using VEIL was comparable to that excised using conventional technology. Of the 9 studies, 2 [27,28] with 52 VEIL procedures used the limb subcutaneous surgical approach (VEIL-L), and 7 studies [29–35] including 197 VEIL procedures used the hypogastric subcutaneous approach (VEIL-H). For patients with intraoperative positive inguinal lymph nodes, 6 studies [28–32,35] with 14 patients performed pelvic lymphadenectomy. Mathevet et al. [28] used the VEIL-L approach and opened for a complete inguinal and external iliac dissection in two cases that were positive on frozen sections. The other 5 studies [29–32,35] used the VEIL-H approach and performed pelvic lymphadenectomy that consisted of moving the trocars and inserting them into the abdominal cavity without creating another incision. Wu et al. [27] included lipolysis and liposuction to expose an adequate operative field and to achieve a cosmetic effect. Coincidentally, Xu et al. [30] used this technique in their previous 10 cases; however, these authors subsequently abandoned that initial technique of fat dissolution and directly used trocar introduction.

**Table 2:** Operative VEIL Parameters.

Author	Operative time, min	Blood loss, ml	Conversion %	Lymph nodes, n	Preserve GSV?	Surgical approach	PLN excision %	Comments
Wu et al. [22]	-	little	0	8.5	YES	VEIL-L	-	Lipolysis and liposuction
Mathevetet al. [28]	62(43–120)	little	2.4	7.5(2–15)	YES	VEIL-L	7.1	-
Cui et al. [24]	80.8	5.5	0	9.5	YES	VEIL-H	13.3	-
Xu et al. [30]	94(70–150)*	137(80–170)^a^	0	16(11–23)*	YES	VEIL-H	29.4	Liposuction
Lu et al. [31]	91(80–130)	6.3(5–10)	0	7.4	YES	VEIL-H	20	-
Liu et al. [32]	83(45–120)	22(10–40)	0	10(6–16)	YES	VEIL-H	25	-
Xia et al. [33]	92.3	6.2	0	7.3	YES	VEIL-H	-	-
Li et al. [34]	102	64.9^a^	0	11.2	YES	VEIL-H	-	-
Tang et al. [35]	110(65–130)	70(40–100)^a^	0	12.8(9–15)	YES	VEIL-H	12.5	-

GSV, great saphenous vein; PLN, pelvic lymph node;

^a^, blood loss containing VEIL and primary tumor excision;

*median value;

-, data not available.

### Postoperative conditions and short-term complications

Table 3 shows the postoperative conditions and short-term complications associated with VEIL. The average/median length of hospital stay varied from 7 to 13.6 days across reports. Li et al. [34] compared 27 patients who underwent traditional open inguinal lymphadenectomy (OIL) with 29 patients who underwent VEIL and reported that the mean length of hospital stay was significantly shorter in the VEIL cases than in the OIL cases (11.6 vs. 17.5 days, *P* = 0.010). This finding was also consistent with a report by Mathevet et al. [28], which included 28 patients who underwent 6 open inguinal lymphadenectomy and 41 VEIL procedures. This study showed that the mean hospital stay after VEIL was only 3.5 days, whereas the overall mean hospital stay was 11 days. The suction drain was removed after 6 to 9.8 days across reports. The postoperative pathological examinations confirmed that 27 (19.7%) patients suffered from lymph node metastasis. One or more short-term complications were documented in 18 of the138 (13.0%) patients. Complications after VEIL were observed in 14 (10.1%) patients and 15 (6.0%) of the VEIL cases, including major lymphocyst formation in 9 (3.6%), lymphorrhea in 2 (0.8%), inguinal wound infection without wound breakdown in 3 (1.2%) and lymphedema in 1 (0.4%). Notably, according to Li et al. [34], the postoperative morbidity was significantly lower in the VEIL group than in the OIL group (*P* < 0.05). In addition, 4 (2.9%) patients developed vulvar wound necrosis.

**Table 3:** General Conditions and Short-Term Complications in the Postoperative Period.

Author	Hospital stay, d	Drain removal, d	LN metastasis, %	Skin-related complications, % Groin infection/ Groin necrosis/ Vulva necrosis	Lymph-related complications, % Lymphorrhea/ Lymphocyst/ Lymphedema
Wu et al. [22]	-	9.8(4–13)	30	0/0/-	0/0/0
Mathevet et al. [28]	11(2–20)	-	14.3	0/0/0	0/17.1/0
Cui et al. [24]	10.7	-	13.3	0/0/6.7	3.3/6.7/0
Xu et al. [30]	11(8–19)*	6(5–8)	29.4	0/0/11.8	0/0/2.9
Lu et al. [31]	-	6.8(5–10)	20	10/0/10	0/0/0
Liu et al. [32]	7(5–12)	-	25	0/0/0	7.7/0/0
Xia et al. [33]	-	6.7	-	0/0/-	-/-/-
Li et al. [34]	11.6	6.7	17.2	3.4/0/0	0/0/0
Tang et al. [35]	13.6	-	12.5	0/0/0	0/0/0

*median value;

-, data not available.

### Survival and recurrence at short-term follow-up

Only 4 studies [28–31] included follow-up with 70 patients after VEIL over a relatively short period (3–41 months). According to these studies, only 3 (4.3%) patients suffered from local recurrence. In addition, the 2 recurrent patients were treated with surgery combined with radiotherapy. Nevertheless, there were no distant metastases and no mortality within the short-term follow-up period.

## Discussion

Inguinal lymph node involvement is an important prognostic factor for lower limb malignant melanoma and lower genital tract neoplasia, such as penile cancer and vulvar cancer. Inguinal lymph node dissection enables the staging and treatment of these diseases. However, traditional open inguinal lymphadenectomy is associated with high morbidity. VEIL was introduced for penile cancer surgery by Tobias et al. in 2006 [37] and was subsequently established as a minimally invasive technique to reduce wound complications while achieving comparable oncological control. With the development of surgical instruments and the enhancement of laparoscopy techniques, single-site VEIL [38] and robot assisted VEIL [39–41] were developed for penile cancer treatment with inguinal lymphadenectomy to increase the minimal invasiveness of the approach. VEIL was subsequently applied to vulvar cancer and limb malignant melanoma. However, there have been no RCTs or large prospective studies on the use of VEIL in vulvar cancer because of the low incidence of this disease. Currently, the majority of VEIL research in vulvar cancer consists of small retrospective studies that preliminarily evaluate safety and feasibility. Thus, we chose to conduct an appropriate evaluation of VEIL by taking previous data into comparison to guide clinicians in the management of vulvar cancer.

### The assessment of the feasibility and safety of VEIL

In our review, various operative parameters including operative time, estimated blood loss and number of harvested lymph nodes were generally acceptable. Particularly, among the intraoperative complications, only 1 case (0.4%) was converted to an open procedure for suturing because of an injury to the femoral vein. Furthermore, these results can be optimized with the development of surgical instruments and enhancement of the laparoscopy techniques.

The short-term complication rate associated with VEIL (10.1% of the patients and 6.0% of the VEIL cases) was quite appreciable, as compared to previously published data (34.1–66%) on traditional inguinal lymphadenectomy [7,42–44]. There are similar complication rates for vulvar cancer patients with traditional inguinal lymphadenectomy: groin necrosis in 6.5–18.8% of patients, groin infection in 5.6–39%, lymph cysts in 1.9–40% and lymphoedema in 28–48.8% [7,42–45]. Morbidity after inguinofemoral lymphadenectomy is impressive despite the type of primary tumor. The high morbidity mainly occurs because a large surgical wound enhances the tension in the tissue surrounding the wound and reduces the blood supply to the inguinal tissue after inguinofemoral lymphadenectomy. There was an obvious decline in the incidence of morbidity in vulvar cancer patients with VEIL: groin necrosis in 0% of patients, groin infection in 0–10% and lymph cysts in 0–17.1%. This result was likely obtained because VEIL is a minimally invasive procedure and the operative incisions kept away from the special inguinal operation area to avoid inguinal skin defects and have a minimal impact on the blood supply to the inguinal tissue. Although the included studies did not analyze the high risk of short-term complications and did not investigate the long-term complications, Lu et al. [31] described a patient with a groin infection and vulva necrosis in the setting of diabetes. Hinten et al. [45] reported that older age, diabetes, ‘en bloc’ surgery and high drain output on the last day of the in situ drain were associated with a higher risk of short-term complications, and younger age and lymphocele were associated with a higher risk of developing long-term complications. However, further research should be conducted to focus on postoperative management.

The reduction of postoperative complications may shorten the length of postoperative hospital stay. Li et al. [34] and Mathevet et al. [28] reported that the length of hospital stay for patients undergoing VEIL was significantly shorter than for patients undergoing traditional open inguinal lymphadenectomy. In our review, 6 studies reported that the mean/average length of postoperative hospital stay (7–13.6 days) was significantly shorter, considering previously published data (11–22 days), than traditional inguinal lymphadenectomy [42,46,47]. This advantage may reduce the economic cost and benefit for undergoing adjuvant therapy such as radiotherapy or chemotherapy.

According to these studies, based on short-term follow-up analysis after VEIL, there were no distant metastases and no mortality. Although the duration of follow-up was short, there was a low recurrence rate (4.3%), particularly compared with the high rate of lymph node metastasis (19.7%). Furthermore, patients with recurrence may undergo surgery combined with radiotherapy. Lu et al. reported that one patient developed local recurrence at the primary site because she did not receive chemotherapy after surgery. Unfortunately, no studies analyzed the high risk of recurrence, and we could not obtain information on patients with disease recurrence regarding whether they had advanced vulvar cancer with lymph node metastases or whether they received adjuvant therapy such as radiotherapy or chemotherapy. Thus, future studies that include long-term follow-up after VEIL are needed to describe the survival and recurrence in greater detail and improve the quality of the evidence for patient outcomes.

### The selection of the surgical approach

There are two surgical approaches for VEIL: a limb subcutaneous approach (VEIL-L) and a hypogastric subcutaneous approach (VEIL-H). Chen et al. [48] conducted a small comparative study on VEIL-L and VEIL-H in vulvar cancer and reported that there was no difference in the short-term outcomes between the two approaches. However, exposure of the operative field when excising deep inguinal lymph nodes was easier with VEIL-L, whereas VEIL-H was more convenient and minimally invasive when conducting pelvic lymphadenectomy. In our review, 2 studies used VEIL-L, and 7 studies used VEIL-H. Patients who underwent VEIL-H benefited from the following comparative advantages. First, bilateral lymphadenectomy was possible through four incisions in the abdominal wall, and this approach avoided additional incisions by moving the trocars and inserting them into the abdominal cavity when pelvic lymphadenectomy was necessary, thereby reducing the number of incisions and operation wounds. Second, there is no wound on the thigh because the drainage tubes exit from the incision in the abdominal wall, and all of the incisions are in the abdominal wall. This could reduce the risk of lower limb lymphedema and dysfunction. Furthermore, the operative time is shortened because there is no need to change the position of the patient during the operation. However, the relative short and long-term outcomes remain unknown.

### Lipolysis and liposuction

Liposuction is one of the most popular procedures in aesthetic plastic surgery [49,50], and this procedure has also been introduced into endoscopic resection of breast cancer auxiliary lymph nodes for exposing the vessels, nerves and lymph nodes, which reduces side injuries, especially to the intercostobrachial nerve. Wu et al. [27] used lipolysis and liposuction and reported that 4 patients developed inguinal skin and subcutaneous tissue hardening. The hardening area returned to elastic skin with a smooth surface after 3 to 5 months when the hypodermic hardened tissue was scattered and absorbed. Although lipolysis and liposuction were beneficial for exposing an adequate operative field and achieving a cosmetic effect, there remains some controversy about this technique. For instance, it seems that the removal of the inguinal lymph nodes from the incision is not consistent with the principle of tumor-free surgical operations. However, if the lymph nodes are positive, it is possible that liposuction could induce tumor metastases locally, although this remains to be determined. Many studies have reported that VEIL should be performed by an experienced team that is skilled not only in laparoscopic techniques but also traditional inguinal lymphadenectomy; there is no need for liposuction if a highly skilled team performs the VEIL procedure. Xu et al. [30] also used liposuction in their initial 10 cases, although these authors subsequently avoided the technique of fat dissolution and directly used trocar introduction.

## Conclusions

VEIL appears to be a feasible approach in the management of vulvar cancer. However, its safety remains to be confirmed by further studies. At present, systematic research is scarce regarding the survival and recurrence in vulvar cancer patients with VEIL, and there are a limited number of publications to date. There may be potential benefits that result in lower morbidity than traditional methods, but this is yet to be objectively proven. Large, multicenters, RCTs are needed, and future research should focus more on postoperative management and follow-up.

## Supporting Information

S1 Fig Flowchart of the Selection Process for Studies Included in the Systematic. Fig 1.
(TIF)

S1 File A copy of PRISMA checklist. (DOC)

S2 File The reasons for exclusion. (DOCX)
