# Supplementary figures and images for: Feasibility and Safety of Video Endoscopic Inguinal Lymphadenectomy in Vulvar Cancer: A Systematic Review
Source: PLoS One. 2015 Oct 23;10(10):e0140873. doi: 10.1371/journal.pone.0140873 (PMC4619862; doi:10.1371/journal.pone.0140873)

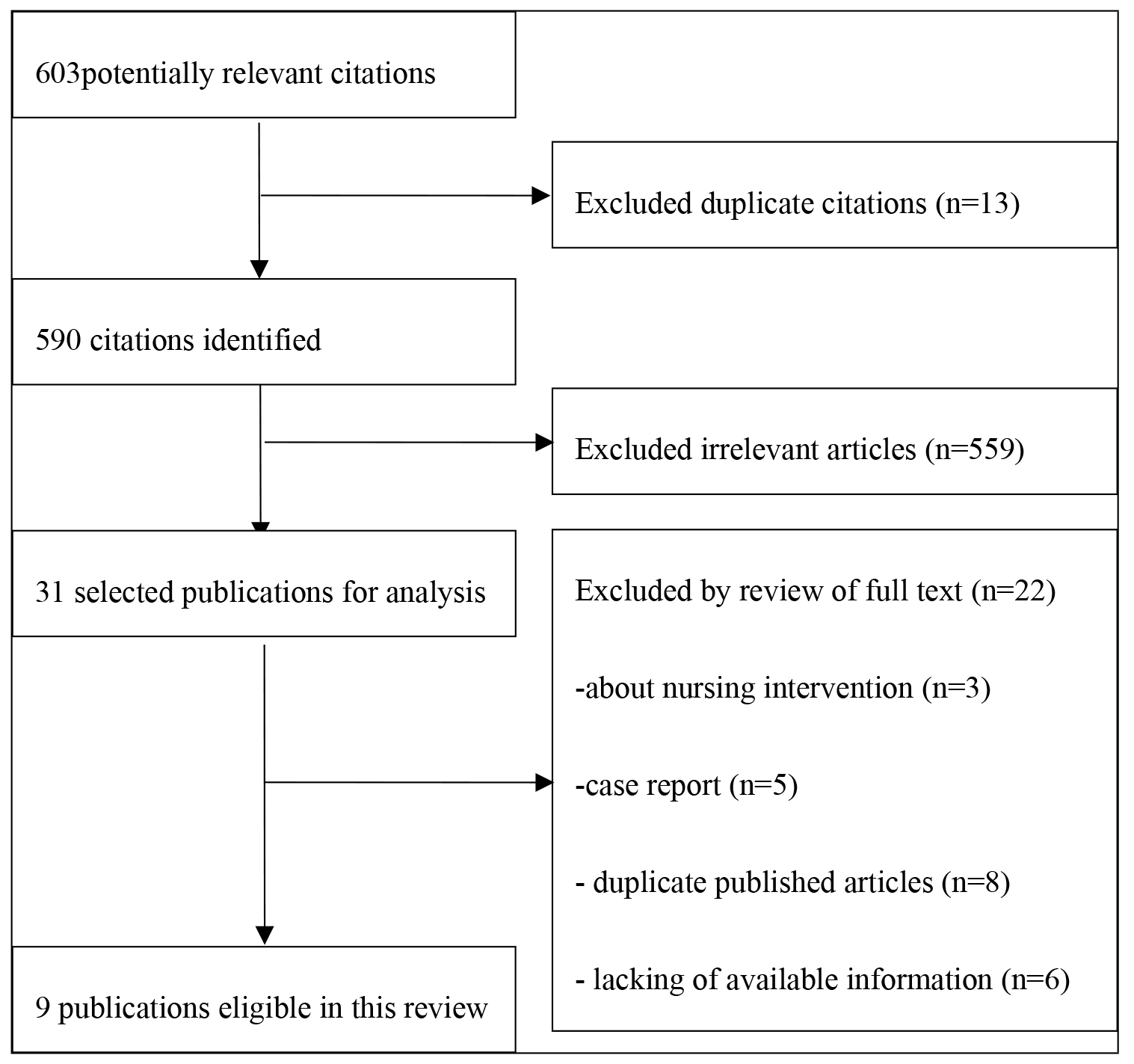

Supplement: S1 Fig — Fig 1. (TIF) [file pone.0140873.s001.tif]
