# Supplementary material for: Feasibility and Safety of Video Endoscopic Inguinal Lymphadenectomy in Vulvar Cancer: A Systematic Review
Source: PLoS One. 2015 Oct 23;10(10):e0140873. doi: 10.1371/journal.pone.0140873 (PMC4619862; doi:10.1371/journal.pone.0140873)
Supplement: S2 File — (DOCX) [file pone.0140873.s003.docx]

**The reasons for exclusion**

After careful selection, 31 publications were selected for the analysis. Of these, 3[1-3] were excluded because they investigated nursing interventions, 5[4-8] were excluded because they were case reports and containing only one vulvar cancer patient. 6[9-14] were excluded because they lacked extractable information regarding VEIL in vulvar cancer patients. In those articles, 5 [9-13] contained not only vulvar cancer, penile cancer, but also epithelioid sarcoma, 1[14] was studying for the position of VEIL and they didn’t analysis the operative parameters or postoperative complications. And 8[15-22] were excluded because they were duplicated published articles. There are 8[5, 7, 9, 11-12, 16-17, 21] articles reported as conference abstracts which were unable to get the full information in the excluded 22 articles.

1. Feng Y, Zhu XL, Wu Q (2012) Nursing intervention cooperated with video endoscopic inguinal lymphadenectomy in vulvar cancer. Chinese General Practice Nursing 10(31): 2919-2920.

2. Xu J (2011) Clinical application of endoscopic lymphadenectomy for vulvar cancer. Journal of Qiqihar University of Medicine 32(11):1786-1787.

3. Zhu MY, Bai TL (2013) Exploration of nursing care cooperated with video endoscopic inguinal lymphadenectomy in vulvar cancer. Medical Information 26(8):490.

4. Huber D, Robyr D, Schneider N (2012) Video endoscopic-assisted inguino-femoral lymphadenectomy (VEIL) in squamous cell invasive vulvar carcinoma: Our initial experience. Gynecol Surg 9(2): 175-178.

5. Huber D, Schneider N (2012) New frontiers of inguinal lymphadenectomy for vulvar cancer: Video-endoscopic approach. Int J Gynecol Obstet 119: S627-S628.

6. Naldini A, Rossitto C, Morciano A, Panico G, Campagna G, et al. (2014) The first leg video endoscopic groin lymphadenectomy in vulvar cancer: A case report. Int J Surg Case Rep 5(8): 455-458.

7. Monagas Arteaga S, Alonso Prieto MA, Garcia Diez F, De Arriba Alonso M, et al. (2013) Initial series of video endoscopic inguinal lymphadenectomy and description of the technique. European Urology, Supplements 12(1): eV60.

8. Ma JJ, Chen BL (2014) Clinical effect and operation strategy on inguinal lymphadenectomy for vulvar carcinoma by using Da Vinci robotic surgical system. Chin J Laparoscopic Surgery (Electronic Edition) (03): 172-176.

9. Goel A, Gupta A, Khanna S, Vashishtha S, Rawal S (2014) Robot assisted video endoscopic inguinal lymphadenectomy: Our single centre experience of complications and outcome analysis. Journal of Urology 191(4): e394-e395.

10. Sudhir R, Krishnappa RS, Khanna S, Sekon R, Koul R (2012) Video endoscopic inguinal lymphadenectomy (VEIL): minimally invasive radical inguinal lymphadenectomy technique. Indian J Surg Oncol 3(3) 257-261.

11. Raghunath SK, Rawal S, Khanna S (2012) Use of video endoscopic inguinal lymphadenectomy (VEIL) in patients with urogenital malignancies: An observational study. Journal of Clinical Oncology 30(15).

12. Sudhir R, Khanna S, Srivatsa N (2012) Robotic assisted video-endoscopic inguinal lymphadenectomy. International Journal of Urology 19: 330.

13. Jiang MQ, Tan J (2013) Laparoscopic pelvic lymphadenectomy in gynecological malignancies: A clinical observation. Modern Journal of Integrated Traditional Chinese and Western Medicine. 22(2): 158-159.

14. Cui W, Bai TL, Wang Y (2014) Research on the improvement of the superior position of the laparoscopic inguinal lymphadenectomy of vulval carcinoma. Medical Information 27(05):477-478.

15. Cui ZY. (2013) The comparison of VEIL-H with OPL in vulvar cancer. Doctoral Dissertations, Southern Medical University. Available: http://muse.jnu.edu.cn:9797/MuseSessionID=9d3fd6fda24fb1aae6815fa45ffd830/MuseHost=d.g.wanfangdata.com.cn/MusePath/Thesis_Y2405904.aspx.

16. Chen LM, Ding JX, Hua KQ (2012) A comparative study about two different inguinal lymphadenectomy in vulvar cancer. Compilation of essays from 10th National Academic Conference of Obstetrics and Gynecology, Chinese Medical Association: 386-387.

17. Liu Q, Han NN, Liu KJ, Ru MF, Li PQ (2013) Clinically application of laparoscope in inguinal lymphdissection of vulvar cancer. Compilation of essays from 6th National Academic Conference of Gynecologic Endoscopy and Minimally Invasive Technology, Chinese Medical Association.

18. Chen G, Wang Y, Wang Y, Zhu H, Wang X, et al. (2014) Video endoscopic inguinal lymphadenectomy via hypogastric/limb subcutaneous approach for early-stage vulvar cancer. National Medical Journal of China 94: 39-42.

19. Wang YF, Chen GW, Weng HN, Sheng XJ, Wong F (2013) Surgical technique of video endoscopic inguinal lymphadenectomy via a hypogastric subcutaneous approach. Chinese Medical Journal 126: 3181-3183.

20. Xu H, Wang Y, Wang D, Li Y, Chen Y, et al. (2010) Laparoscopic inguinal lymphadenectomy for the vulvar cancer: Description of technique and surgical outcomes. Journal of Minimally Invasive Gynecology 17: S111.

21. Liu Q, Han NN, Li PQ, Liu KJ (2012) Clinically application of laparoscope in inguinal lymphdissection of vulvar cancer. Compilation of essays from 10th National Academic Conference of Obstetrics and Gynecology, Chinese Medical Association: 180.

22. Xu HC, Wang YZ, Li YD, Wang D, Chen Y, et al. (2011) Laparoscopic inguinal lymphadenectomy: a new minimally invasive technique to treat vulva cancer. Chinese Journal of Practical Gynecology and Obstetrics (04): 283-285.
